# Early phases of sepsis: effects of simvastatin on mitochondrial enzyme activities in kidney tissue in rats

**DOI:** 10.1186/cc14060

**Published:** 2014-12-03

**Authors:** E Özkök, H Yorulmaz, G Ateş, İ Aydın, AŞ Tamer

**Affiliations:** 1 Department of Neuroscience, The Institute for Experimental Medicine, Istanbul University, Istanbul, Turkey; 2 School of Health of Science, Halic University, Istanbul, Turkey; 3 Department of Physiology, Istanbul Medical Faculty, Istanbul University, Istanbul, Turkey; 4 Department of Histology and Embryology, Istanbul Medical Faculty, Istanbul University, Istanbul, Turkey

## Introduction

Acute kidney injury (AKI) is a frequent and serious complication of sepsis. Moreover, there is strong evidence that AKI in patients with severe sepsis is associated with a higher mortality rate. The devastating effects of Gram-negative sepsis are largely based on the effects of lipopolysaccharide (LPS), also known as endotoxin. Mitochondrial dysfunction has been suggested to contribute to the development of organ dysfunction and failure in sepsis. The mitochondrial electron transport chain consists of four complexes (CI to CIV) and its function can be assessed with different approaches. Statins, such as simvastatin and atorvastatin, are hypocholesterolemic drugs that possess pleiotropic effects, including antioxidant and anti-inflammatory properties, that are either dependent on or independent of 3-hydroxy-3-methyl-glutaryl-CoA reductase (HMG-CoA reductase) inhibition. In addition, *in vitro *and *in vivo *studies have demonstrated that simvastatin has an anti-inflammatory effect in patients with predialytic chronic kidney disease, and may play an important role in counteracting the mechanisms involved in pathogenesis of inflammation. We aimed to investigate the effects of prior simvastatin on mitochondrial enzyme activities in kidney tissue of the early phase of sepsis.

## Methods

We used male adult Wistar albino rats weighing 200 to 250 g in the experiments. The rats were divided into four groups, each composed of eight rats: control group, LPS group, Simvastatin group, Simvastatin + LPS group. Lipopolysaccharide (LPS) from *Escherichia coli *O127:B8 (Sigma, St. Louis, MO, USA) was injected intraperitoneally at a daily dose of 20 mg/kg, Simvastatin (20 mg/kg) was given p.o. via oral gavage for 5 days. In the Simvastatin + LPS-treated group, LPS was given 1.5 hours after the fifth dose of simvastatin. Mitochondrial electron transport chain enzymes citrate synthase, NADH-cytochrome c reductase (complex I + III), and NADH dehydrogenase (complex I) were measured kinetically in spectrophotometer from kidney tissue homogenate. The kidney tissue samples were fixed in 10% buffered formalin and embedded in paraffin for hematoxylin and eosin (H&E) staining. Data were expressed as mean ± standard deviation (SD) and analyzed during analysis of variance.

## Conclusion

**Figure 1:**
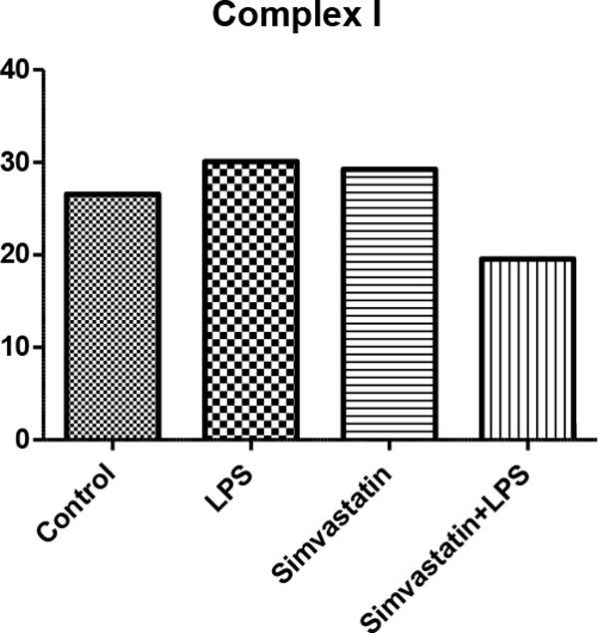
**Complex I activity (µmol/minute/g tissue)**.

There were no changes in activities of citrate synthase, complex I, complex I + III in tissue homogenate (*P *>0.05) (Figures 1 to Figure 3). In the LPS + Simvastatin group, we found decreased activity of complex I compared with those of the LPS and Simvastatin groups (*P *= 0.05, *P *= 0.07, respectively). As a result of the light microscopic examination with H&E stained sections, we observed tubule lumens widened and partially damaged in the epithelium. In the Simvastatin group was seen partially widened tubular structure and even in some areas tubular structure was found the same as control sections. Moreover, the damage in tubular width and proximal epithelium was observed to continue (Figure 4).

**Figure 2:**
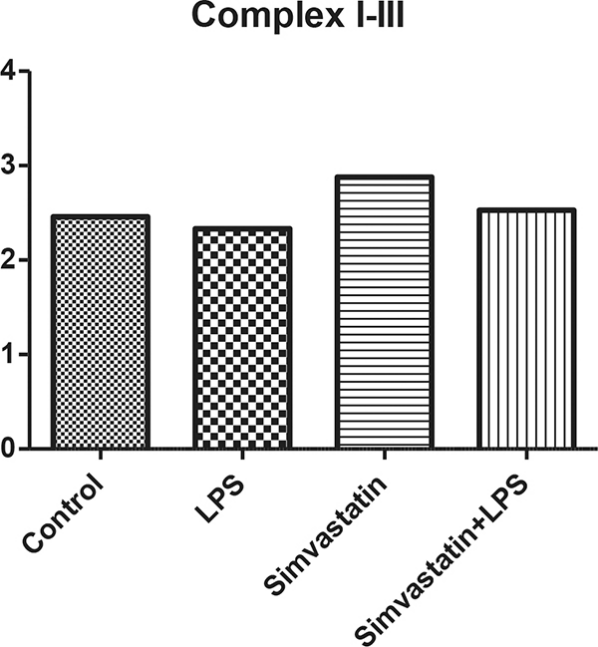
**Complex I + III activity (µmol/minute/g tissue)**.

**Figure 3:**
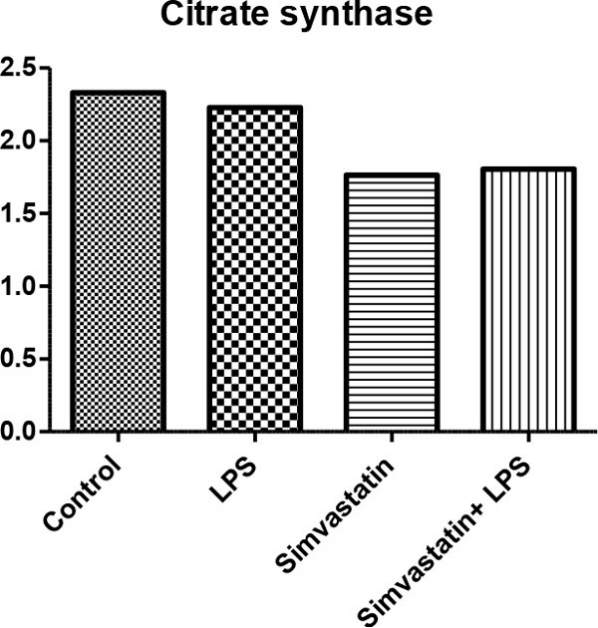
**Citrate synthase activity (µmol/minute/g tissue)**.

**Figure 4:**
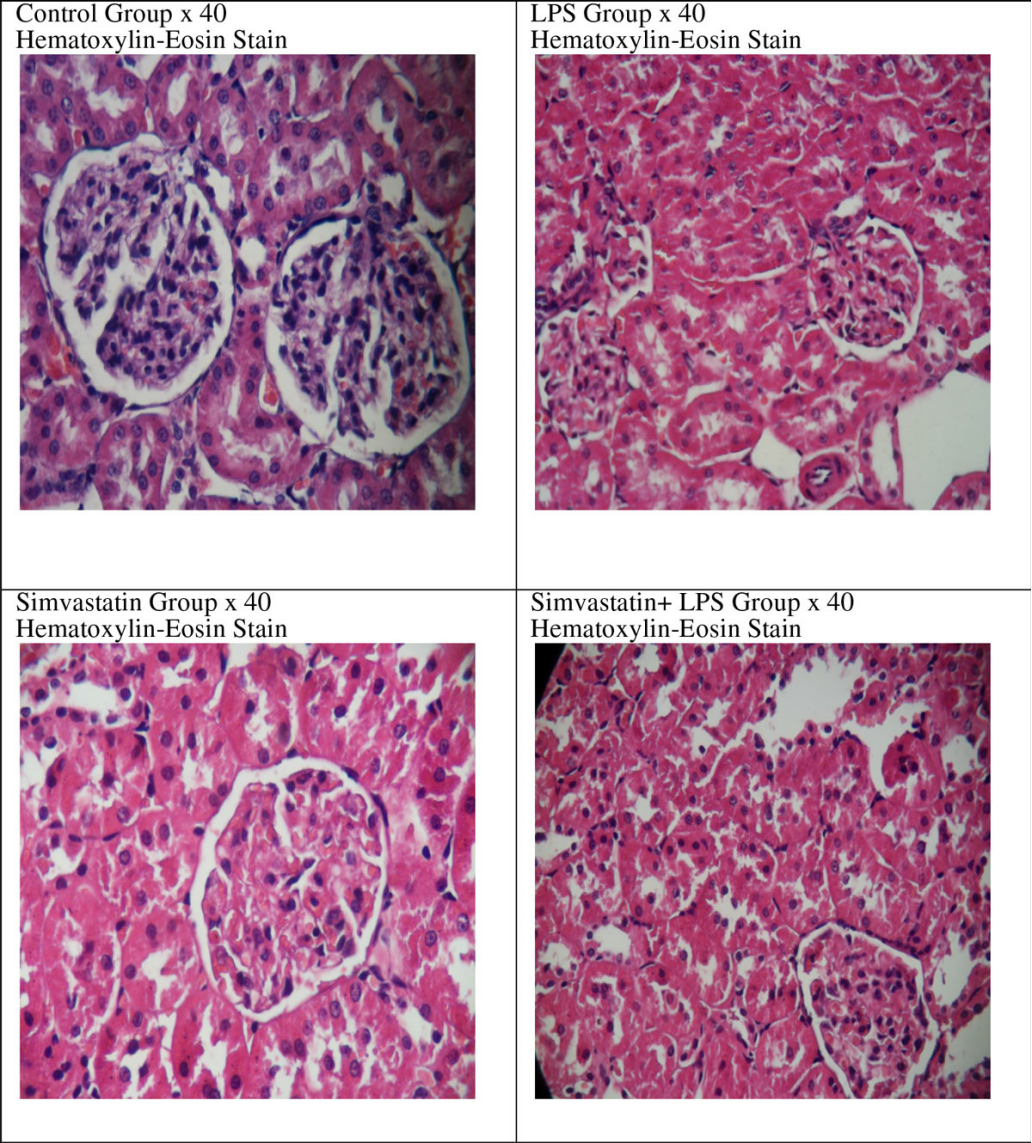
**Hematoxylin and eosin staining in all experimental groups**.
